# Supplementary figures and images for: Tensor decomposition of multi-dimensional splicing events across multiple tissues to identify splicing-mediated risk genes associated with complex traits
Source: PLoS Comput Biol. 2025 Jul 21;21(7):e1013303. doi: 10.1371/journal.pcbi.1013303 (PMC12303388; doi:10.1371/journal.pcbi.1013303)

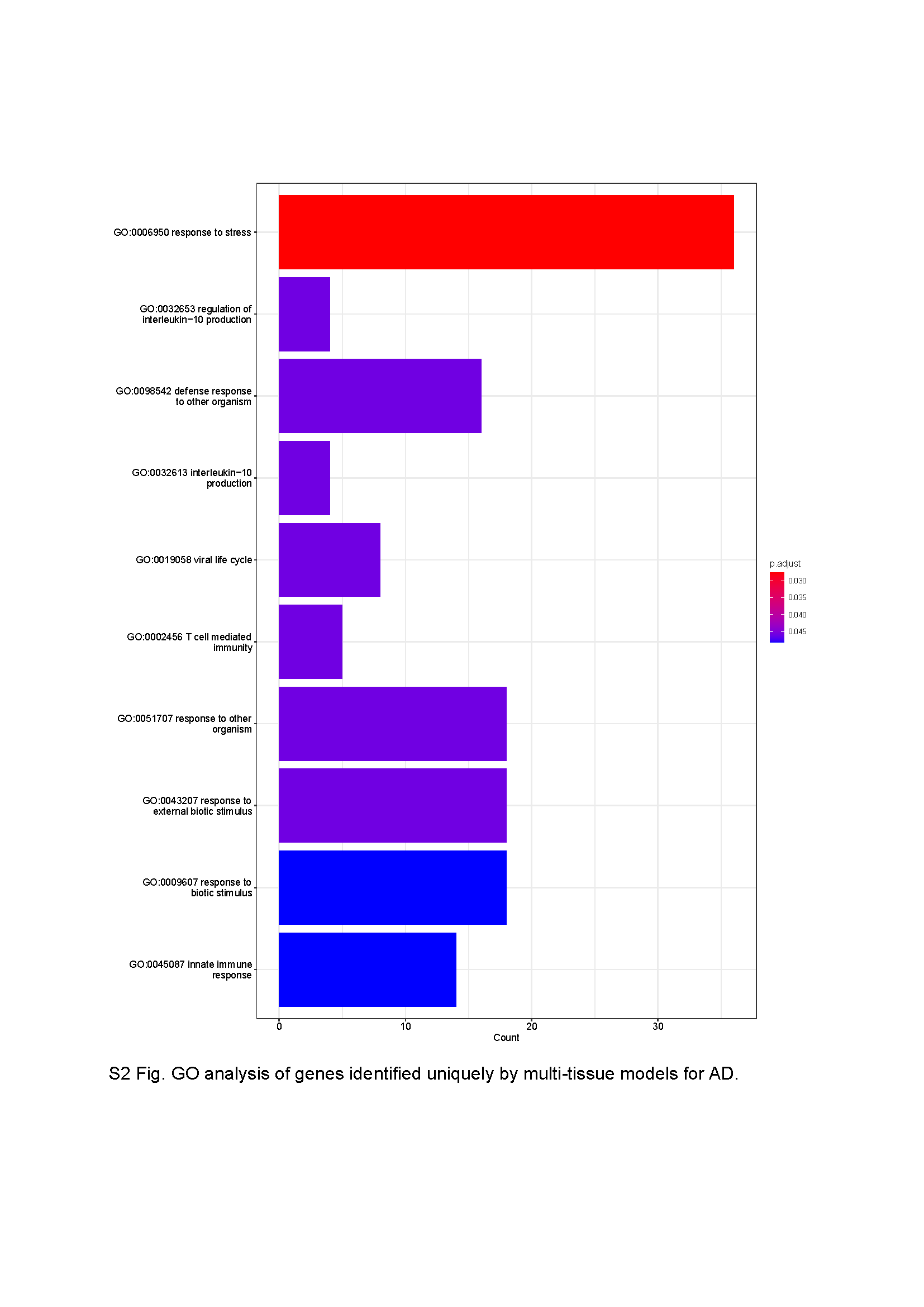

Supplement: S2 Fig — (TIF) [file pcbi.1013303.s002.tif]

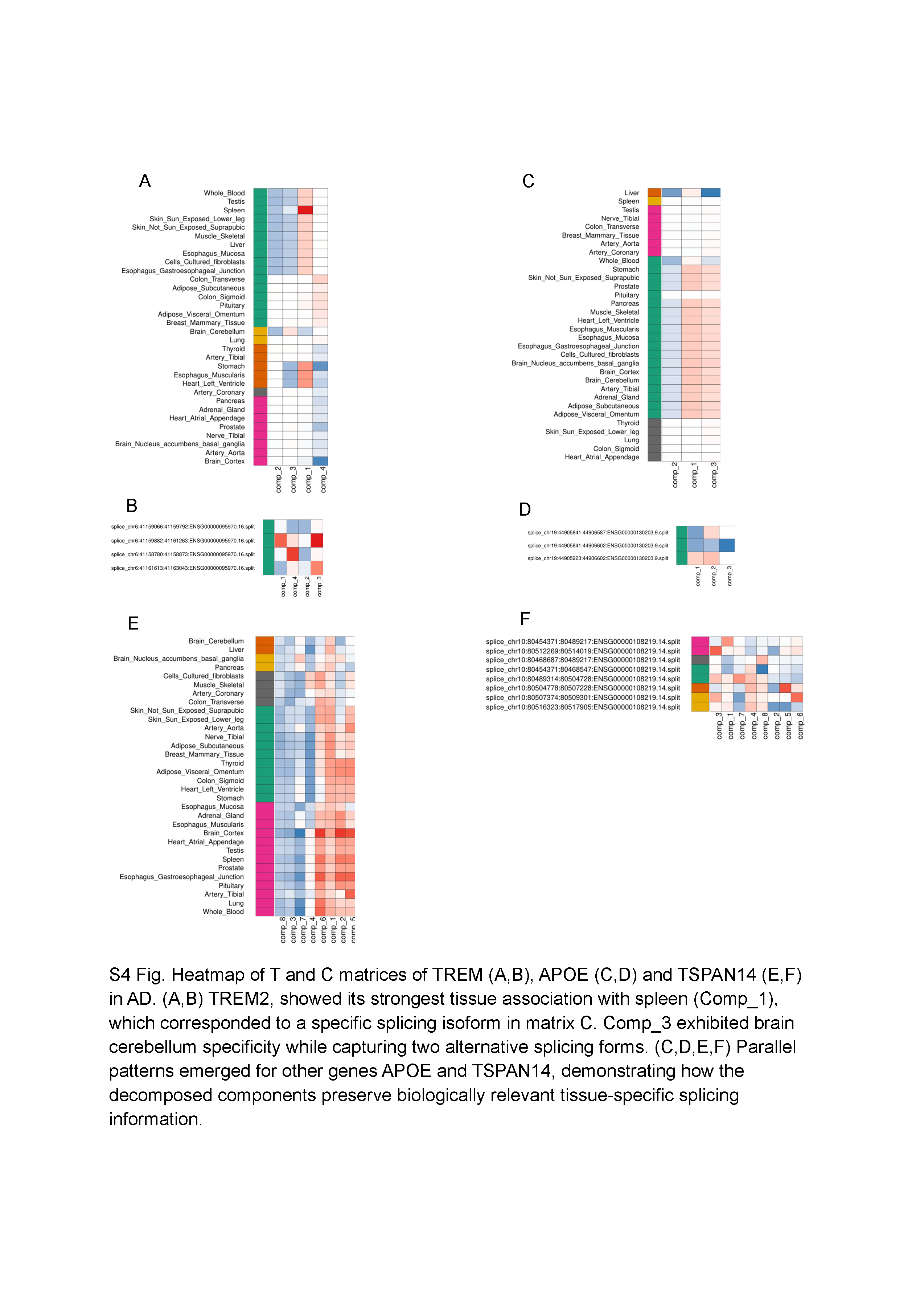

Supplement: S4 Fig — (A,B) TREM2, showed its strongest tissue association with spleen (Comp_1), which corresponded to a specific splicing isoform in matrix C. Comp_3 exhibited brain cerebellum specificity while capturing two alternative splicing forms. (C,D,E,F) Parallel patterns emerged for other genes APOE and TSPAN14, demonstrating how the decomposed components preserve biologically relevant tissue-specific splicing information. (TIF) [file pcbi.1013303.s004.tif]

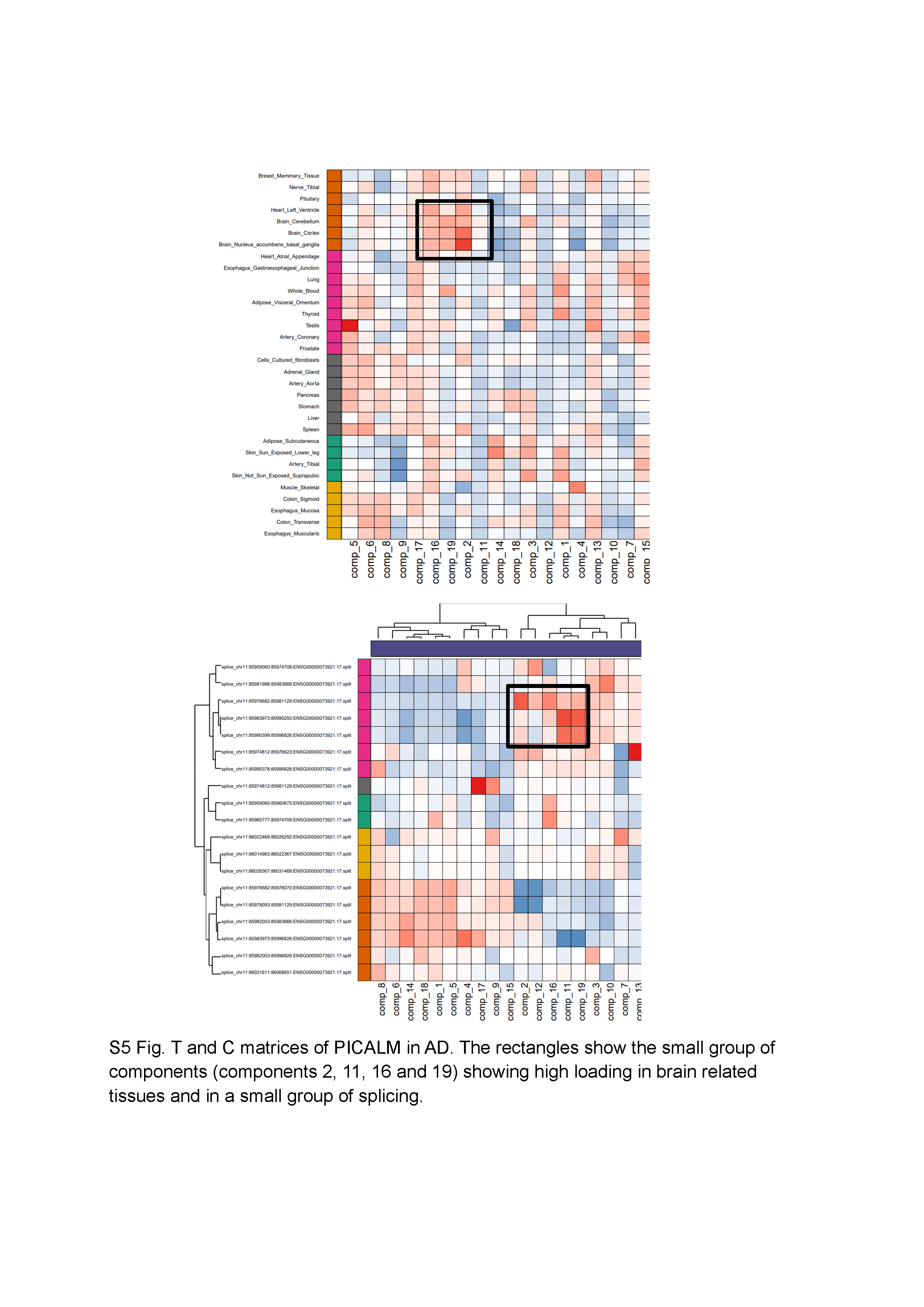

Supplement: S5 Fig — The rectangles show the small group of components (components 2, 11, 16 and 19) with high loading in brain related tissues and in a small group of splicing. (TIF) [file pcbi.1013303.s005.tif]

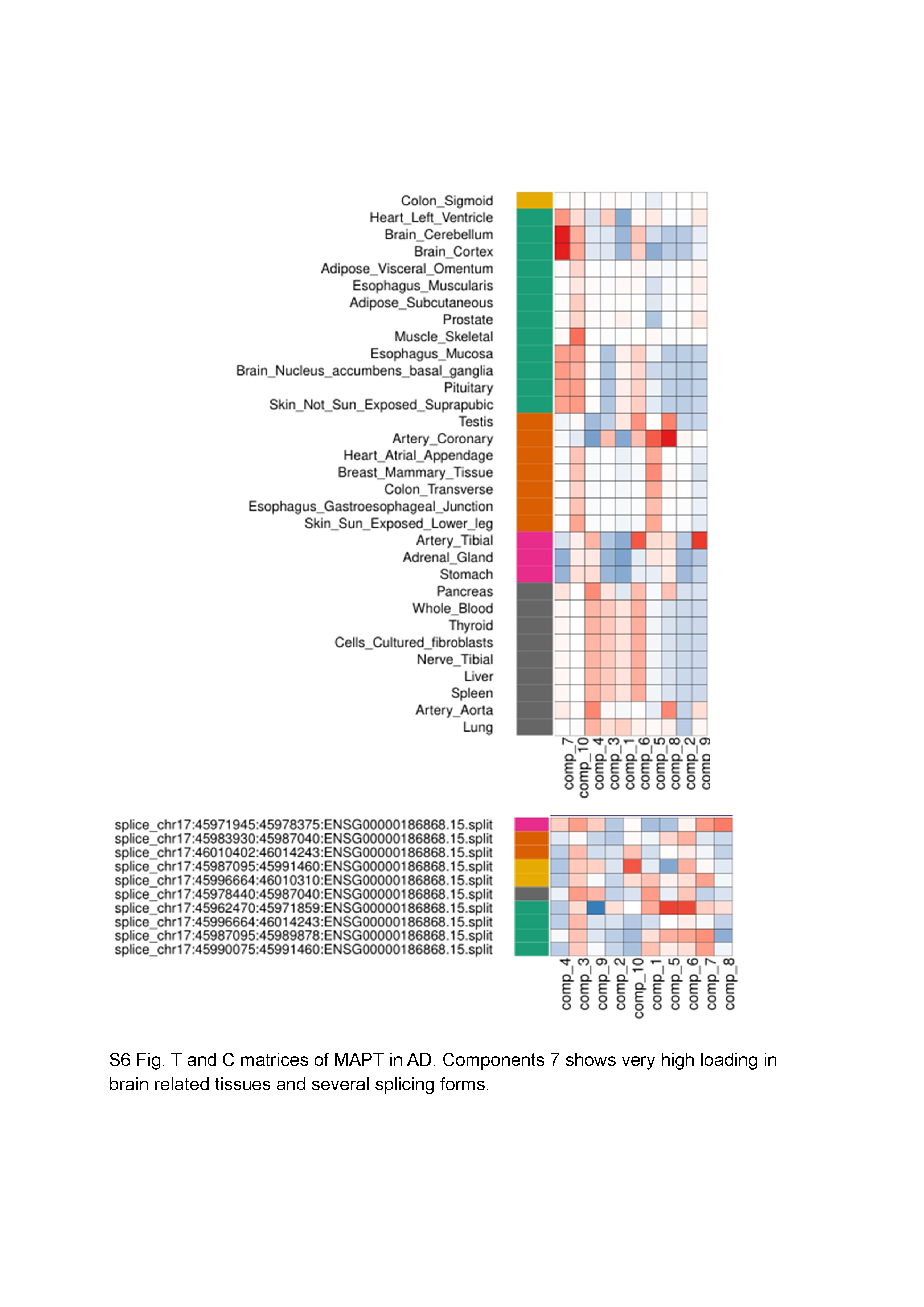

Supplement: S6 Fig — Components 7 shows very high loading in brain related tissues and several splicing forms. (TIF) [file pcbi.1013303.s006.tif]

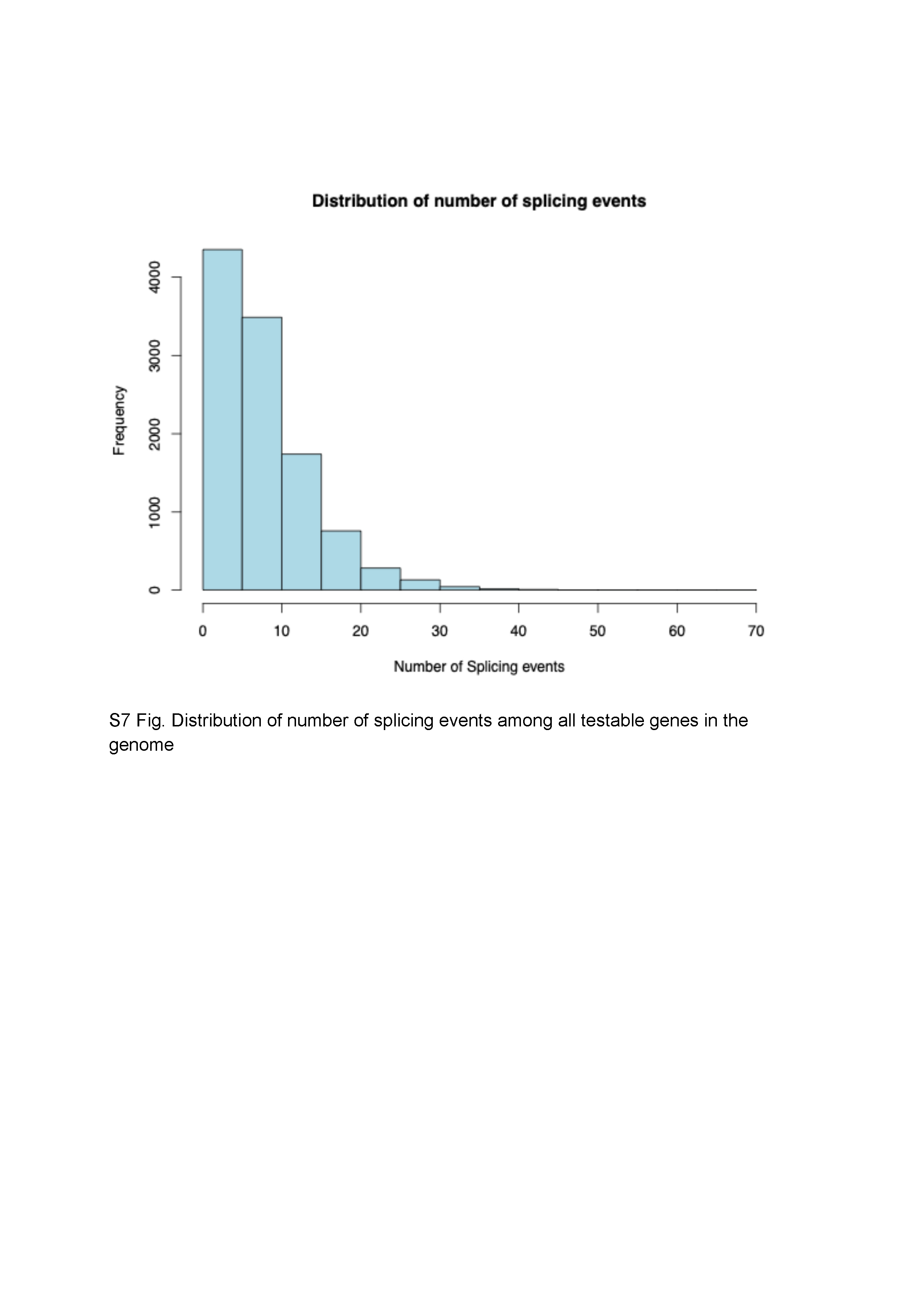

Supplement: S7 Fig — (TIF) [file pcbi.1013303.s007.tif]
