# Supplementary material for: Tensor decomposition of multi-dimensional splicing events across multiple tissues to identify splicing-mediated risk genes associated with complex traits
Source: PLoS Comput Biol. 2025 Jul 21;21(7):e1013303. doi: 10.1371/journal.pcbi.1013303 (PMC12303388; doi:10.1371/journal.pcbi.1013303)
Supplement: Attachment — Submitted filename: MTSG_response_to_reviewers.docx [file pcbi.1013303.s014.docx]

**Reviewers' comments:**

**Reviewer's Responses to Questions**

**Reviewer #1: The Multi-Tissue Splicing Gene (MTSG) model, utilizing tensor decomposition and sparse Canonical Correlation Analysis (sCCA), effectively integrates multi-tissue splicing event data to identify splicing-mediated risk genes associated with complex diseases such as Alzheimer's Disease (AD) and Schizophrenia (SCZ). This innovative approach not only demonstrates the potential of multi-tissue models in improving the identification of disease-related genes but also enhances the statistical power compared to single-tissue models. The genes identified by the model are more relevant to the genetic etiology of these diseases, and the multi-tissue analysis captures splicing events that may be overlooked in single-tissue studies, thereby providing valuable insights for personalized medicine. In total, this work makes a valuable contribution by developing the MTSG model, significantly enhancing the identification of splicing-mediated risk genes in complex diseases. Here are some concerns:**

1. I am curious about the difference between the results obtained using individual tissue and those obtained by integrating all tissues, because the paper demonstrates **the advantage of using multiple tissue data to increase the number of identified genes and the statistical power of the analysis**. To quantify this difference, maybe the authors could calculate the Jaccard index for the genes identified across different tissues, or something like that. This would allow you to **assess the overlap and consistency between the results from individual tissues versus the multi-tissue analysis.** Such an analysis would help in understanding the contribution of different tissues to the disease identification process and further validate the reliability of MTSG model.

A: We thank the reviewer for their valuable suggestion to quantify the differences between single-tissue and multi-tissue analyses. Following the recommendation, we calculated the Jaccard Index (JI) to assess the overlap between gene sets identified by MTSG and individual tissues. The analysis revealed a JI of 0.358 between MTSG and BA9, with values ranging from 0.272 to 0.36 across all 32 tissues. This narrow JI range indicates that MTSG captures signals distributed more or less evenly across tissues rather than being biased toward specific tissues, while still preserving tissue-specific variations.

We add a description to the main text (page 10, paragraph 1):

*“**The Jaccard Index (JI), a similarity metric for comparing two sets, ranges from 0 (no overlap) to 1 (complete identity). Our analysis revealed a JI of 0.358 between the gene sets of MTSG and BA9. Furthermore, we computed JIs between MTSG and all 32 individual tissues, with values ranging from 0.272 to 0.36 (Fig. S1). This relatively narrow range suggests that the signals detected by MTSG are distributed evenly across the tissues.”*

2. The authors mention that the number of components in the tensor decomposition was chosen based on the number of splicing events for each gene. **It would be helpful to include an analysis to show how different choices of the number of components might impact the results.** This analysis could help readers understand the potential influence of parameter choices on the identified risk genes and their biological relevance.

A: We appreciate the reviewer’s insightful question regarding the influence of the number of components (NC) in tensor decomposition. While determining the optimal NC remains challenging in practice, we systematically evaluated its impact by re-running analyses with alternative NC values (0.5× and 1.5× our original choice, NC_1). For NC_1.5, we identified 190 genes (63.7% overlapping with NC_1 results), while NC_0.5 yielded 94 genes (71.3% overlapping). Notably, ~85% of NC_0.5 genes and ~79% of NC_1.5 genes ranked within the top 300 of NC_1 results, demonstrating robust preservation of top-ranked genes despite p-value variations. We also rewrote the theoretical considerations about why we specify the number of components to the number of splicing events for each gene. Together, these analyses confirm that our primary findings are robust to NC selection, with consistent prioritization of biologically relevant genes across parameter choices.

We add a description to our manuscript (Page 24, Paragraph 4):

*“The selection of the rank is supported by the following considerations: First, our model is theoretically well-defined given the specified r (which is set to p), in the sense that it is uniquely identifiable according to the Kruskal uniqueness condition (53) for tensor factorization. Specifically, in our data, the number of splicing events is often small and always below the number of subjects (median = 8, as shown in Supplementary Figure S7). This yields that the sum of the rank of subject, splicing event, and tissue loading matrices is 2p+t or 3p, whichever is the smaller. This will always be larger than 2p+2, which, according to Kruskal et al., is sufficient to guarantee the uniqueness of the model’s factorization formulation. Second, our approach generates a comprehensive representation of genes’ possible splicing event patterns, ready to be utilized in the downstream sCCA analyses. By aligning r with the number of splicing events, we will be able to represent all possible splicing combinations for each gene (in its splicing event loading matrix). The corresponding subject loading matrix will thus provide a comprehensive profile of each subject’s splicing configurations, for sCCA to effectively extract predictive patterns between SNPs and splicing components. Third, we conducted additional analyses using two alternative rank thresholds: 0.5 and 1.5 times of p, comparing with the results presented here. The results demonstrate that approximately 80% of the genes identified under these alternative thresholds were consistently ranked in the top 3% genes in our primary analysis. These variations in gene identification can be attributed to differences in p-value distributions across the threshold settings. In this way, we ensure that our tensor decomposition is theoretically sound, biologically interpretable, computationally feasible, and optimized for downstream analyses.”*

3. The authors impute missing data in the GTEx dataset using the "mice" R package. Have the authors tested different imputation methods, and how might the choice of imputation technique influence the results? It would be useful to include **a brief discussion on the potential limitations or biases introduced by imputation**, especially when dealing with large-scale multi-tissue data.

We appreciate the reviewer's important question regarding imputation effects. We sincerely appreciate the reviewer for catching this oversight. There was indeed a typographical error in our original manuscript where we incorrectly specified the imputation method. For clarity, we used the Amelia package for data imputation in our analysis, not the method initially stated. We have now carefully corrected this throughout the manuscript to ensure accuracy. We appreciate this valuable feedback, which has helped improve the precision of our methodological description.

To address this, we rigorously evaluated two established imputation methods - MICE (Multivariate Imputation by Chained Equations) and Amelia - selected for their proven performance with high-dimensional biological data. MICE's flexibility with mixed data types (continuous splicing ratios and categorical covariates) and Amelia's strength in cross-sectional data made them ideal candidates. Our systematic comparison involved introducing 10% random missingness to the complete data subsets and calculating RMSE between imputed and observed values. Amelia demonstrated superior accuracy (RMSE=0.18 vs 0.24 for MICE), leading to its selection for downstream analyses. We further mitigated the bias by only retaining splicing events present in ≥12 tissues. While traditional methods like k-nearest neighbors struggle with multi-tissue data scalability, we acknowledge emerging deep learning approaches (e.g., hypergraph factorization) may offer future improvements.

We add a paragraph to Discussion (Page 23, Paragraph 2):

*“For association testing across multiple tissues, imputation is necessary due to varying sample availability across different tissues. The accuracy of this imputation step may introduce additional variability. We evaluated two widely used imputation packages: MICE (Multivariate Imputation by Chained Equations) (49) and Amelia. Using a subset of complete data with simulated 10% random missingness, we compared the root mean square error (RMSE) between imputed and observed values. Amelia demonstrated superior performance with a lower RMSE (0.18 vs. 0.24 for MICE), indicating better reconstruction accuracy, and was consequently selected for downstream analyses. While other methods (e.g., k-nearest neighbors) may not scale effectively for multi-tissue splicing data or handle mixed data types as robustly, recent advances in deep learning have shown promising results for multi-tissue data imputation (50,51). Future improvements could incorporate these state-of-the-art deep learning approaches.”*

4. While the manuscript effectively identifies splicing-mediated risk genes, it would be helpful if the authors could further explore the **biological impact of specific splicing events** on gene function in the **discussion section**. For instance, the authors could attempt to explain how certain splicing events might alter the structure or function of the resulting protein products and contribute to disease pathogenesis. Understanding the molecular mechanisms by which these splicing events affect gene function could provide deeper insights into their role in complex traits like AD and SCZ. Additionally, examining whether certain splicing events are associated with changes in protein domains or the formation of isoforms with distinct functional properties would enhance the manuscript's biological interpretation and impact.

We appreciate the reviewer's important question regarding biological impact of the significant genes. Our framework is building on sCCA, which is jointly testing association between two matrices. The sCCA first finds two vectors *w* and *u* to maximize the correlation between the SNP matrix and the component matrix and then tests the rhos calculated from *w* and *u* pairs to get the significance. The *u* vectors as loadings can be used for identifying the contributions of each component. After getting interesting components, we can map them to corresponding tissues and spicing using C and T loading matrices to get a potential explanation. However, in our methods, we next use the vector *w* for SNP matrix and test the association of gene use *w* and *z*-statistics from summary statistics. This means the statistics are from an overall and we cannot exactly know the contribution of each component. This strategy is more like a group-wise test method aggregating the signals from all components together.

We searched the literature to identify studies focused on splicing-mediated genes in AD, and added a new section (“*Genes with Splicing-Mediated Mechanisms in AD”)* to describe a few genes in terms of their biological function and the potential mechanisms mediated by splicing.

We acknowledge that alternative splicing remains a relatively understudied area in the context of neurodegenerative diseases, largely due to the complexity of the problem in nature. Given the current limitations in both available data, it is challenging to provide further in-depth analysis regarding how specific splicing events might affect protein domains or generate functionally distinct isoforms. Through the above analyses, it is reassuring that our framework has indeed identified genuine splicing-mediated risk genes, with orthogonal support from other studies. As more comprehensive splicing datasets become available in the future, we anticipate that more detailed mechanistic interpretations will be possible.

We hope this clarification adequately explains our current position and the scope of this study. To address the reviewer's valuable suggestions to the extent possible with our current data, we have incorporated a new section in the manuscript that includes the following analyses (Page 15, paragraph 3):

“*Genes with Splicing-Mediated Mechanisms in AD*

*We conducted a literature review to confirm that our MTSG approach effectively captures splicing-mediated risk genes in Alzheimer's disease and provides meaningful biological insights. For example, APOE, one of the most well-characterized AD risk genes, exhibits functionally consequential alternative splicing in its intron-3 region, which modulates both expression levels and clinical severity in AD (36). For the MAPT gene that encodes tau proteins, alternative splicing produces six major tau isoforms with developmentally regulated expression. Altered expression and aggregation of these isoforms underlie AD (37). Our analysis of its T matrix demonstrates components showing strong loadings specifically in brain tissues that correspond to multiple distinct splicing isoforms (S5 Fig.). Notably, Raj et al.'s study (38) conducted a comprehensive analysis of aberrant splicing patterns incorporating intronic excision levels in Alzheimer's disease (AD), and identified 16 genes associated with AD. Our MTSG results show significant overlap with Raj et al.'s gene set (P = 1×10−9), including five overlapping genes: FUS, MTCH2, PICALM, PTK2B, and RABEP1. For example, PICALM is involved in both the production and clearance of amyloid-β (Aβ), a principal component of amyloid plaques in AD pathogenesis (39). Multiple PICALM isoforms are expressed in human brain tissue, and overall PICALM expression levels - particularly of the major isoform - show significant correlation with AD risk (40). Our T matrix analysis corroborates these findings, revealing a subset of components with high loadings specifically in brain tissues that correspond to distinct splicing variants (S6 Fig.). All others have supportive evidence showing clear implicate in AD (41-44).”*

5. Small typos in the manuscript:
“After repeating the procedure p-1 times we obtain p sets of weights (w1, w2)…”, it should be “(w1, u1)” in the page 21.

We corrected it.

“Recently TWAS has been extended to joint modeling of multiple tissues and showed improved powe than single tissues in identifying trait associated genes.”, it should be “power” in the page 5.

We corrected it.

**Reviewer #2: In the manuscript “Tensor decomposition of multi-dimensional splicing events across multiple tissues to identify splicing-mediated risk genes associated with complex traits”, the author described a framework called MTSG, which extracts latent factors in mRNA splicing across multiple contexts (e.g. tissues) by tensor decomposition, followed by CCA analysis with genotype data to build a predictive model. This model was then applied to GWAS summary statistics to identify disease-associated genes that potentially mediate disease etiology through splicing. The authors showed that this method is better powered than canonical single-tissue analysis and could be extended to study other diseases and molecular phenotypes. In general, although the method and data analysis in this manuscript look promising, the authors mostly reported high-level results like the number of significant genes and gene-set enrichment analysis. Therefore, I would like to see more details of the tensor decomposition results and how this method compared to other methods more commonly used to link genes to disease GWAS loci.**

Major comments:
1. Tensor decomposition is central to the method proposed in this manuscript. But little detail was given about its performance. Instead, the authors directly jump to sCCA analysis and interpretation of gene association results. I would like to see more about the results from tensor decomposition:
a. Specifically, I’m curious about what **matrices C and T** look like and whether they could provide meaningful information as well. For example, **does matrix T show tissue-specific pattern of the components**; **does matrix C show which splicing events are driving each topic**?

We appreciate the reviewer's question regarding the interpretation of our tensor decomposition results, and performed additional analysis on how C and T matrices are able to reveal further insights for AD. Overall, the results showed that both the tissue (T) and splicing (C) matrices provide valuable insights about the tissues and splicing events beyond the gene-trait associations.

The tissue matrix T shows that whole blood (53 genes), brain cerebellum (50 genes), brain cortex (44 genes), brain nucleus accumbens basal ganglia (43 genes), and liver (41 genes) exhibit the strongest loadings for all significant genes. These patterns remain largely consistent for MTSG-specific genes, while the overlapping genes with BA9 show some variation, with pancreas and cultured fibroblasts appearing among the top tissues. Importantly, the prominence of immune-related (whole blood) and lipid metabolism-enriched (liver) tissues aligns well with established AD pathogenesis (36,37).

The splicing matrix C demonstrates how specific splicing events drive each component. For instance, TREM2 shows its strongest association with Component 1 in spleen, corresponding to a particular splicing isoform in C, while Component 3 exhibits brain cerebellum specificity capturing two alternative splicing forms. Similar patterns emerge for other AD-associated genes like APOE and TSPAN14, where specific splicing events consistently associate with tissue-specific components. These results collectively demonstrate that our tensor decomposition not only identifies gene-trait associations but also captures biologically plausible tissue-specific expression patterns and meaningful relationships between splicing events and tissue contexts, supporting the validity of our approach. We have incorporated these interpretations in the revised manuscript to better highlight these important findings.

We added a section to the manuscript (Page 15, Paragraph 1) – thanks to the reviewer’s suggestion:

*“*

*Biological Interpretation of the Splicing Matrix C and Tissue Matrix T*

*While the sample matrix Y serves for gene-trait association testing, we focus on extracting biological insights from the splicing matrix C and tissue matrix T. By ranking tissues based on their loadings in matrix T, we identified whole blood (53 genes), brain cerebellum (50 genes), brain cortex (44 genes), brain nucleus accumbens basal ganglia (43 genes), and liver (41 genes) as the top contributors of significant genes (Fig. S3A). This tissue distribution pattern remained largely consistent for genes uniquely identified by MTSG, while the overlapping genes between MTSG and BA9 showed some variation, with pancreas and cultured fibroblasts appearing in the top ranks (Fig. S3B). Notably, the prominence of immune-related whole blood and lipid metabolism-enriched liver tissues aligns with known AD pathology (36,37).*

*At the gene level, we observed distinct tissue and splicing specificity patterns. TREM2, a well-characterized AD risk gene, showed its strongest tissue association with spleen (Component 1), corresponding to a specific splicing loading in matrix C (Fig. S4A, B), and Component 3 exhibited brain cerebellum specificity while capturing two alternative splicing forms. Similar patterns emerged for other AD-associated genes like APOE and TSPAN14, demonstrating how the decomposed components preserve biologically relevant tissue-specific splicing information (Fig. S4C-F). These findings collectively highlight the utility of tensor decomposition not only for association detection but also for uncovering tissue-specific regulatory mechanisms underlying disease. “*

2. The authors compared the MTSG method with single-tissue method, **using only one tissue in their comparative analysis (brain frontal cortex BA9)**. A likely scenario is that some splicing events are tissue-specific, so they might not be found in brain frontal cortex BA9. This might explain why MTSG tend to find more genes than single-tissue method. **A fairer comparison would be to run single-tissue analysis in all tissues included in the MTSG model and take the union set of significant genes across all tissues**.

We thank the reviewer for raising this important point. To address it, we conducted single-tissue analyses for each of the tissues, identifying 416 significant genes (79-135 per tissue). The results showed that 42% (175/416) of genes were tissue-specific (detected in only one/two tissues), while the majority of genes shared by MTSG and single tissues (111/131) are likely to be identified by more than one tissue. Importantly, MTSG uniquely detected 47 genes, including established AD risk genes (APOE, BIN1), demonstrating its ability to capture robust, multi-tissue signals while filtering tissue-specific noise. At the same time, the key AD genes like APOE and BIN1 were missed by single-tissue analyses in any of the tissues, indicating the reduced power of single-tissue analyses. While we considered the union approach, we retained BA9 as our primary comparison due to its biological relevance to AD and to avoid noise from non-relevant tissues. These findings are now summarized in the manuscript (Page 10, Paragraph 2):

*“We also compared the MTSG with all single-tissue analyses. Our single-tissue analyses identified 416 significant genes after multiple testing correction, with each tissue contributing 79-135 genes. Notably, 175 genes (42%) exhibited strong tissue specificity, being detected in only one or two tissues, likely enriched for false positives, considering that tissues often have shared regulatory mechanisms. In contrast, the majority of genes (111/131) identified by both MTSG and individual tissues showed cross-tissue consistency, supporting our method's ability to capture biologically robust signals through cross-tissue information integration. Importantly, MTSG uniquely detected 47 genes, including established AD risk genes (APOE, BIN1), demonstrating its ability to capture robust, multi-tissue signals while filtering tissue-specific noise. “*

3. The authors argue that MTSG finds more genes while preserving most top genes in single-tissue analysis (Abstract). But this is not immediately clear by reading the main text: “In total, MTSG identified 174 significant genes after Bonferroni correction at 5% level (Supplementary Table 3 for the list of genes), while the single tissue models identified 160, with 88 genes in common” (line 156-158) and “We conducted a separate analysis focusing solely on the brain frontal cortex BA9, which revealed 476 significant genes. Among them, 292 genes were common to both datasets…” (line 237-238). These numbers suggest that the overlap between MTSG and single-tissue analysis is only about 50%, meaning that the two methods often find very different genes. Therefore, a better comparison of the two gene lists is needed. Maybe the authors could break down the gene lists by certain criteria to **support the notion that MTSG can “preserving most top genes in single-tissue analysis**”.

We appreciate the reviewer's careful examination of the gene overlap between MTSG and single-tissue analyses. While the nominal overlap is approximately 50%, a more nuanced rank-based analysis reveals stronger concordance. Specifically, among the 147 MTSG-identified genes testable in BA9, 81% ranked within BA9's top 300 genes and 85% in the top 500. Conversely, 76-85% of BA9's 134 significant genes ranked in the top 500 of our multi-tissue results. This demonstrates that while the absolute overlap appears modest, MTSG consistently preserves the most statistically significant single-tissue findings while identifying additional high-confidence genes through cross-tissue integration. These findings validate that MTSG maintains high sensitivity to top single-tissue signals while providing enhanced detection power, addressing the reviewer's concern about methodological comparability. The rank-based comparison provides more meaningful evaluation than simple overlap percentages, as it accounts for differences in statistical power and multiple testing thresholds between approaches.

As detailed in our revised manuscript (Page 10, Paragraph 2):

*“We also explore how MTSG preserves the signals identified by single tissue. Our analyses demonstrate strong concordance between MTSG and single-tissue results, with approximately 50% overlap in significant genes. Notably, when examining the 147 significant genes identified by MTSG that were testable in BA9, we observed that 81% ranked within the top 300 BA9 genes, and 85% within the top 500. Conversely, the majority of BA9's 134 significant genes (76% and 85%) similarly ranked within the top 300 and 500 genes, respectively, in our multi-tissue analysis. These results robustly demonstrate that MTSG effectively preserves the many significant genes from single-tissue analyses while enhancing detection power through cross-tissue integration.“*

4. For AD analysis, it is unclear where the increased power come from compared with single-tissue analysis. **Is it because MTSG shares and aggregates information across tissues, or is it because there’s some tissue-specific splicing**? I would like to see more analysis on the genes unique to MTSG in AD analysis. **Could some signals come from non-brain tissues (AD GWAS colocalize with immune QTL a lot)**? If yes, **what's the interpretation**? Also, related to interpretation, what is the PSI for the introns in MTSG-unique genes across tissues? Can the T or C matrices be used to help answer these questions?

We thank the reviewer for raising important questions about the source of MTSG's increased power and the biological interpretation of our findings. Below we provide a comprehensive responses addressing each concern:

The first question is: “**Is it because MTSG shares and aggregates information across tissues, or is it because there’s some tissue-specific splicing**?”

As addressed in question 2, the enhanced power of MTSG stems from both identifying genes with cross-tissue effects while also detecting genes that cannot be tested in individual tissues. Among the 86 MTSG-specific genes, 11 were untestable in BA9 alone, including several with prior disease associations. For example, BCL3 was implicated in AD through rare variant analysis near APOE (PMID: 28589856), while PPP1R13L emerged from hippocampal-focused TWAS and demonstrated cross-tissue effects across at least three tissues (PMID: 33630843). This demonstrates MTSG's ability to integrate information across tissues. While single-tissue analyses can detect genuine signals, particularly in causal tissues, they risk identifying tissue-specific genes with lower signal-to-noise ratios as evidenced by our GO analysis. MTSG provides complementary value by capturing these cross-tissue associations that might otherwise be missed, while reducing false positives compared to single tissues when the tissues are not directly relevant to the disease.

The second question is: “**Could some signals come from non-brain tissues (AD GWAS colocalize with immune QTL a lot). what's the interpretation**?”

Analysis of the tissue matrix (T) revealed that the top contributing tissues for MTSG-specific genes include Whole_Blood, Brain_Cerebellum, Brain_Cortex, Brain_Nucleus_accumbens, and Liver, collectively covering 70 of the 86 MTSG specific genes. The prominence of immune-related whole blood and lipid metabolism-enriched liver tissues aligns with AD's known non-CNS pathology (PMID: [36351425](https://pubmed.ncbi.nlm.nih.gov/36351425/); PMID: [34997690](https://pubmed.ncbi.nlm.nih.gov/34997690/)), suggesting that these peripheral tissues contribute biologically plausible signals beyond traditional brain-centric mechanisms.

The next question is: “**what is the PSI for the introns in MTSG-unique genes across tissues?**”

The Percent Spliced In (PSI) metric quantifies intron inclusion levels but requires raw splicing count data, which is unavailable in the public GTEx dataset. For the reviewer’s questions, we did not calculate PSI in this study, and are therefore not able to provide the PSI for the introns for these genes. In our current data analysis, it is not straightforward to calculate PSI, as it requires processing raw sequencing data from scratch, which would involve extensive efforts, including quality control, batch effect correction, and computational effort. While PSI could reveal extreme intron inclusion differences, our normalized splicing matrices (C and T) already capture tissue-specific splicing patterns, as demonstrated in question 1. Given that our current approach provides partially comparable tissue-specific splicing information, we hope that our responses are able to address the reviewer’s comment, at least partially. We appreciate the reviewer’s further advice on this if the reviewer considers PSI analysis is essential for generating substantially new insights beyond what our matrices already reveal.

The last question is: “**Can the T or C matrices be used to help answer these questions?**”

As highlighted throughout our responses (question 1), matrices T and C provide a powerful lens for interpreting results. Matrix T identifies tissue-specific contributions (e.g., brain vs. non-brain tissues), while matrix C reveals the splicing isoforms driving each component. Together, they enable biologically coherent interpretations, such as *APOE*'s and *TSPAN14*'s tissue- and splicing-specific patterns.

We have integrated these insights into the revised manuscript, including a new table summarizing top contributing tissues and expanded discussion of immune/lipid pathways. These additions strengthen our interpretation while maintaining methodological rigor. We appreciate the opportunity to clarify these points. Please refer to our responses in question 1 for further details.

5. Brain tissues are known to have more complex splicing patterns, which are more likely to benefit from MTSG method. **I'm curious if MTSG could be applied to other GWAS summary statistics as a comparison, such as rheumatoid arthritis, crohn's disease, T2D or height, all of which have high-quality public GWAS data. Of course I'm not suggesting the authors should do all of these.**

We appreciate the reviewer's insightful suggestion regarding the broader applicability of MTSG. Our framework is indeed generalizable to other diseases. Following the reviewers’ suggestion, we applied MTSG to additional diseases, including HDL (183 genes), inflammatory bowel disease (419 genes), major depressive disorder (462 genes), and rheumatoid arthritis (323 genes). In each case, the identified genes showed strong enrichment in biologically relevant pathways (e.g., cholesterol transport for HDL, cytokine signaling for IBD/RA, and neuronal pathways for MDD), validating our method's generalizability.

While these results demonstrate MTSG's versatility, we focused on AD and SCZ in this manuscript for several reasons: (1) They represent distinct categories of brain disorders - AD with its microglia- and lipid-driven pathology versus SCZ as a neurodevelopmental neuron-focused disorder; (2) They showcase our method's ability to handle different disease mechanisms; and (3) They align well with our team's core expertise, ensuring rigorous interpretation. Although we successfully applied MTSG to other diseases, we feel that comprehensively dissecting each of the diseases would unnecessarily digress from the central theme of this study. Instead, we present the genes we identified for each of the diseases in Supplemental data to the research community, and briefly describe the findings in the manuscript – see below. We hope that this addition addresses the reviewer's concern while maintaining our focus on the most biologically informative examples. The consistent performance across diverse traits underscores MTSG's robustness as a general framework for splicing-mediated disease gene discovery.

As noted in our revised Discussion (Page 24, Paragraph 2):

“*Our framework demonstrates broad applicability for identifying splicing-mediated risk genes across diverse complex traits. Successful applications to HDL (64) (183 genes), IBD (65) (419 genes), MDD (66) (462 genes), and RA (67) (323 genes) have revealed disease-relevant pathways that align with established pathological mechanisms, confirming the generalizability of our approach (Table S4).*”

Minor comments:
1. In SCZ analysis, only brain tissues were used. What is the rationale? Please explain if possible.

For SCZ analysis, we restricted our investigation to brain tissues based on overwhelming evidence of CNS-specific pathogenesis of schizophrenia. Three key findings support this approach: (1) tissue enrichment analyses consistently identify neuronal cells as primary mediators of SCZ genetic risk (PMID: 29632380); (2) co-regulation patterns show strongest disease association in glutamatergic/GABAergic neurons (PMID: 37580597); and (3) single-cell studies confirm neuronal specificity of SCZ signatures (PMID: 36175791). This brain-focused strategy enhances the power to detect neuronal splicing variants while reducing noise from peripheral tissues, as evidenced by our pathway analyses revealing strong synaptic/neuronal enrichment among identified genes. The approach aligns with SCZ's neurodevelopmental origins and prevailing disease models.

2. Typo on line 81, should be “improved power”, not “improved powe”.

We corrected it.

**Reviewer #3: This manuscript explores the identification of splicing-mediated risk genes using multi-tissue splicing data and tensor decomposition. While the approach is methodologically interesting, the study lacks sufficient biological justification and interpretability. My detailed comments are as below.**

1. The manuscript argues that splicing information provides a finer resolution for identifying risk genes compared to gene expression. However, it does not provide a clear mechanistic rationale for how splicing, rather than gene expression, is functionally linked to the traits examined.

Thank you for raising this important question regarding the mechanistic rationale for focusing on splicing. While gene expression studies (eQTL/TWAS) have significantly advanced our understanding of disease mechanisms, we emphasize splicing analysis because it accounts for comparable heritability as gene expression in human diseases (Page 4 Paragraph 1). Our approach is motivated by findings like those from the Alzheimer's study (PMID: 30297968), which identified 87 intron clusters (84 genes) with altered splicing, of which only 11 showed differential expression - demonstrating splicing can reveal associations missed by expression analysis alone. Our method aims to identify splicing-trait associations, analogous to sQTL or splicing-TWAS approaches. To enhance biological interpretation, we've added analysis showing how our tensor decomposition extracts tissue-specific splicing patterns. For example, we found whole blood, brain regions and liver as top contributors, aligning with AD pathogenesis. Specific genes like TREM2 showed spleen-associated splicing isoforms, while APOE and TSPAN14 exhibited brain-specific splicing patterns. This demonstrates how our approach preserves biologically relevant tissue-specific splicing information for downstream mechanistic studies.

We added this section to the main text (Page 15, paragraph 1):

*“Biological Interpretation of the Splicing Matrix C and Tissue Matrix T*

*While the sample matrix Y serves for gene-trait association testing, we focus on extracting biological insights from the splicing matrix C and tissue matrix T. By ranking tissues based on their loadings in matrix T, we identified whole blood (53 genes), brain cerebellum (50 genes), brain cortex (44 genes), brain nucleus accumbens basal ganglia (43 genes), and liver (41 genes) as the top contributors of significant genes (Fig. S3A). This tissue distribution pattern remained largely consistent for genes uniquely identified by MTSG, while the overlapping genes between MTSG and BA9 showed some variation, with pancreas and cultured fibroblasts appearing in the top ranks (Fig. S3B). Notably, the prominence of immune-related whole blood and lipid metabolism-enriched liver tissues aligns with known AD pathology (36,37).*

*At the gene level, we observed distinct tissue and splicing specificity patterns. TREM2, a well-characterized AD risk gene, showed its strongest tissue association with spleen (Component 1), corresponding to a specific splicing loading in matrix C (Fig. S4A, B), and Component 3 exhibited brain cerebellum specificity while capturing two alternative splicing forms. Similar patterns emerged for other AD-associated genes like APOE and TSPAN14, demonstrating how the decomposed components preserve biologically relevant tissue-specific splicing information (Fig. S4C-F). These findings collectively highlight the utility of tensor decomposition not only for association detection but also for uncovering tissue-specific regulatory mechanisms underlying disease. “*

This question importantly connects to Reviewer 1's Question 4. In our response to that previous query, we have provided detailed explanations regarding how the splicing events identified by our MTSG method correlate with disease traits, including specific examples and mechanistic insights. We would respectfully direct the reviewer to that comprehensive response (Reviewer 1 Question 4) for a more thorough treatment of this topic. We hope this cross-referencing approach will help demonstrate both the consistency of our findings and our sincere commitment to addressing all reviewer questions with substantive, evidence-based responses. We also added a part to the manuscript:

“*Genes with Splicing-Mediated Mechanisms in AD*

*We conducted a literature review to confirm that our MTSG approach effectively captures splicing-mediated risk genes in Alzheimer's disease and provides meaningful biological insights. For example, APOE, one of the most well-characterized AD risk genes, exhibits functionally consequential alternative splicing in its intron-3 region, which modulates both expression levels and clinical severity in AD (38). For the MAPT gene that encodes tau proteins, alternative splicing produces six major tau isoforms with developmentally regulated expression. Altered expression and aggregation of these isoforms underlie AD (39). Our analysis of its T matrix demonstrates components showing strong loadings specifically in brain tissues that correspond to multiple distinct splicing isoforms (Fig. S5). Notably, Raj et al.'s study (40) conducted a comprehensive analysis of aberrant splicing patterns incorporating intronic excision levels in Alzheimer's disease (AD), and identified 16 genes associated with AD. Our MTSG results show significant overlap with Raj et al.'s gene set (P = 1×10−9), including five overlapping genes: FUS, MTCH2, PICALM, PTK2B, and RABEP1. For example, PICALM is involved in both the production and clearance of amyloid-β (Aβ), a principal component of amyloid plaques in AD pathogenesis (41). Multiple PICALM isoforms are expressed in human brain tissue, and overall PICALM expression levels - particularly of the major isoform - show significant correlation with AD risk (42). Our T matrix analysis corroborates these findings, revealing a subset of components with high loadings specifically in brain tissues that correspond to distinct splicing variants (Fig. S6). All others have supportive evidence showing clear implicate in AD (43–46).*”

2. The manuscript claims that multi-tissue splicing analysis adds value by leveraging cross-tissue splicing patterns, yet it fails to provide any evidence supporting this claim. A key advantage of multi-tissue data is the ability to identify tissue-specific splicing patterns or risk genes; however, the authors do not present results demonstrating such patterns. Specifically, the study does not highlight which tissues contribute most to the associations, nor does it compare tissue-specific effects to single-tissue models. Furthermore, the identified risk genes are not shown to exhibit distinct splicing patterns across tissues.

We appreciate the reviewer's thoughtful questions and address each concern below:

The first question is: “**A key advantage of multi-tissue data is the ability to identify tissue-specific splicing patterns or risk genes; however, the authors do not present results demonstrating such patterns.”**

We acknowledge that conventional multi-tissue approaches are valuable for identifying tissue-specific splicing patterns through individual tissue analyses. While these tissue-specific signals are biologically important given the tissue-dependent nature of splicing, our analyses reveal that focusing exclusively on them presents challenges. As shown in our response to Reviewer 2 Question 2, single-tissue analyses often yield numerous genes specific to just one or two tissues, many of which may represent noise or be difficult to interpret due to inclusion of biologically irrelevant tissues: “The results showed that 42% (175/416) of genes were tissue-specific (detected in only one/two tissues), while the majority of genes shared by MTSG and single tissues (111/131) are likely to be identified by more than one tissue. Importantly, MTSG uniquely detected 47 genes, including established AD risk genes (APOE, BIN1), demonstrating its ability to capture robust, multi-tissue signals while filtering tissue-specific noise.”

Our MTSG approach addresses this by identifying splicing patterns shared across multiple tissues, building on established findings of shared local expression regulation across tissues (PMID: 28343628). Importantly, we view our method as complementary rather than superior to tissue-specific analyses - both approaches provide distinct but valuable insights. The strength of our framework lies in its ability to capture these cross-tissue patterns while maintaining the capacity to identify tissue-specific effects through our decomposition approach.

The second question is: “**Specifically, the study does not highlight which tissues contribute most to the associations”**

We thank the reviewer for this important question regarding tissue contributions, which complements our response to Reviewer 2 Question 1. Our analysis of the T matrix revealed the top contributing tissues for the 86 MTSG-specific genes to be Whole_Blood (53 genes), Brain_Cerebellum (50), Brain_Cortex (44), Brain_Nucleus_accumbens_basal_ganglia (43), and Liver (41), collectively accounting for 70 of the 86 genes. These findings are presented in detail in our Results section (Page 15, paragraph 1), where we highlight several biologically meaningful patterns: the prominence of immune-related whole blood and lipid metabolism-enriched liver tissues aligns with known AD pathology, while at the gene level we observe specific associations such as TREM2's spleen-specific isoform, Component 3's brain cerebellum-specific splicing forms, and consistent tissue-specific patterns for other AD genes including APOE and TSPAN14. These results robustly demonstrate how our tensor decomposition approach effectively captures both cross-tissue patterns and biologically relevant tissue-specific information.

This revision has already presented in question 1.

The next question is**: “nor does it compare tissue-specific effects to single-tissue models.”**

The choice between single-tissue and cross-tissue association approaches involves important trade-offs that reflect fundamental biological principles and methodological considerations.

Single-tissue analyses offer superior interpretability for cell context-dependent gene functions, as gene regulation and splicing patterns are often highly tissue-specific. This approach is particularly valuable when studying diseases with well-defined tissue pathology (e.g., neuronal genes in brain disorders). However, its major limitation emerges when analyzing tissues unrelated to the disease of interest, which may introduce substantial noise and false positives.

Cross-tissue approaches address these limitations while capitalizing on shared regulatory architecture. Given the tissue-dependent nature of transcription regulation and the presence of shared regulations across tissues, joint modeling can significantly enhance performance. Several innovative methods have been developed: MultiXcan tests joint effects of gene expression variation across tissues; Sparse-CCA-TWAS combines predicted expression from multiple tissues; and UTMOST employs penalized multivariate regression with group-lasso penalties to identify cross-tissue eQTLs.

While these methods improve prediction accuracy over single-tissue approaches, they have important limitations. Cross-tissue approaches preferentially identify associations shared across all tissues, potentially missing biologically relevant effects present in single tissues. Our recommendation is to run the single tissue association if relevant tissue is available, and run multi-tissue models if relevant tissues are unclear or when the goal is to identify shared signals shared by all or a subset of tissues. As an example, APOE is not identified by any single tissue model but is identified by MTSG.

Additionally, the MTWAS method (PMID: 38982044) incorporates both shared and tissue-specific expression signals, partitioning and aggregating cross-tissue and tissue-specific genetic effects to identify gene-trait associations. It should be noted that this method does not specifically focus on splicing. Developing an integrated framework that includes splicing analysis could be a valuable future direction. For researchers with specific hypotheses, examining single-tissue gene lists may provide additional insights.

The last question is **“Furthermore, the identified risk genes are not shown to exhibit distinct splicing patterns across tissues.”**

We appreciate the reviewer's valuable observation. Our analysis of the T and C matrices for all significant genes reveals that the components effectively capture distinct splicing patterns with tissue-specific distributions. As detailed in our response to Reviewer 2 Question 1 and presented in the main text (Page 15, paragraph 2), these patterns demonstrate clear biological relevance:

*“At the gene level, we observed distinct tissue and splicing specificity patterns. TREM2, a well-characterized AD risk gene, showed its strongest tissue association with spleen (Component 1), corresponding to a specific splicing loading in matrix C (Fig. S4A, B), and Component 3 exhibited brain cerebellum specificity while capturing two alternative splicing forms. Similar patterns emerged for other AD-associated genes like APOE and TSPAN14, demonstrating how the decomposed components preserve biologically relevant tissue-specific splicing information (Fig. S4C-F). These findings collectively highlight the utility of tensor decomposition not only for association detection but also for uncovering tissue-specific regulatory mechanisms underlying disease. “*

3. The manuscript employs tensor decomposition to extract latent components from multi-tissue splicing data, yet it does not leverage the biologically meaningful information embedded in the splicing event-specific and tissue-specific loadings. These loadings provide critical insights into how splicing variations manifest across tissues and how they contribute to trait associations, but the authors do not utilize them in their downstream analysis. Instead, they rely solely on sample loadings without justifying why splicing- or tissue-specific information was excluded. This omission represents a significant loss of potentially valuable biological insights, which is not adequately explained in the study.

We sincerely thank the reviewer for this insightful suggestion. While our method's primary aim is to test gene-trait associations by examining correlations between components and genotypes (hence our initial focus on sample loadings), the reviewer's suggestion to analyze tissue and splicing loadings has proven exceptionally valuable. Following this recommendation, we have conducted comprehensive analyses of the T and C matrices for significant genes and incorporated key examples into the main text (as detailed in our response to Question 1 and 2). These new analyses have substantially enhanced the biological interpretability of our results, providing important insights into tissue-specific splicing mechanisms that would otherwise have remained unexplored. The reviewer's constructive feedback has truly strengthened our study by uncovering additional layers of biological meaning in our data. We are deeply grateful for this contribution that has made our manuscript more complete and impactful.

4. The manuscript assumes that missing values in splicing data arise from technical artifacts and require imputation. However, splicing event absence in a particular tissue may represent a true biological zero rather than a technical dropout. The impact of this assumption should be examined, as imputing biologically meaningful zeros could introduce biases into the analysis.

Thanks for pointing out this important question. This makes us notice that we haven’t explained the imputation very clearly.

In our analysis of splicing data, we carefully distinguished between and handled two types of missing values differently. The first type represents biologically absent splicing events (biological zero), where certain splicing variants are genuinely not expressed in specific tissues. For instance, when examining Gene A with five potential splicing variants (s1-s5) across tissues, we might observe that Tissue X expresses s1, s2, s4, and s5 while lacking s3, and Tissue Y expresses s1-s4 but not s5. In such cases of biological absence, we conservatively filled in these missing values using the minimum observed value across the splicing matrix as this best approximates biological zero expression levels.

The second type involves technical missing data resulting from incomplete individual coverage across tissues. For example, when studying Gene A across five individuals (i1-i5), Tissue X might have data for individuals i1-i4 while Tissue Y covers i1-i3 and i5. The resulting missing measurements (such as i5's data in Tissue X or i4's in Tissue Y for certain splicing variants) represent technical gaps that required statistical imputation. This dual approach of distinguishing between biological absence and technical missingness ensures our analyses properly account for both the biological reality of tissue-specific splicing patterns and the technical limitations of sample coverage while maintaining the integrity of our downstream multi-tissue comparisons.

To address the reviewer’s concern, we have added a description in the method section (Page 27, Paragraph 2):

*“To handle missing values in the splicing data, we implemented distinct approaches for two types of missingness. For biologically absent splicing events (where variants are not expressed in certain tissues), missing values were imputed using the minimum observed value across the splicing matrix as a conservative estimate approaching biological zero. For technical missing data resulting from incomplete individual coverage across tissues, we performed statistical imputation to account for gaps in sample measurements. This dual strategy ensures appropriate treatment of both biological reality and technical limitations while maintaining data integrity for multi-tissue analyses.”*

5. Several methodological aspects lack sufficient clarity:
- The LD matrix is mentioned but not defined in the main text or figure captions.

Thanks for pointing out this error. We fixed it by adding an explanation:

“LD matrix (Linkage Disequilibrium matrix) is a symmetric matrix that quantifies the correlation between genetic variants (e.g., SNPs) due to their co-inheritance patterns.”

Please refer to Fig. 1 legend.

- The definition of p (the number of components) is ambiguous. It appears to refer to the number of splicing events per gene, but this should be explicitly stated.

Thanks for the correction. We have revised the text to make it clear (Page 24, Paragraph 4):

*“For a given gene, we can construct the third-order tensor* ***𝒢*** *of size n x r x t, where n, r, and t denote the number of individuals, the number of components, and the number of tissues, respectively. The number of components r is set to p (number of splicing events for each gene).”*

- Page 19, line 360 contains an incomplete sentence, suggesting that some methodological details may be missing.

Thank you for this important observation. We had planned to address the issue of component number selection in our discussion. This topic was also raised by Reviewer 1 in Question 2. We have now conducted a thorough examination of how different component configurations impact our results and have explicitly discussed these findings. We deleted the incomplete sentence. Accordingly, we have updated the Methods section to provide a more detailed explanation of this crucial aspect of our analysis (Page 24, paragraph 4).

“*The selection of the rank is supported by the following considerations: First, our model is theoretically well-defined given the specified r (which is set to p), in the sense that it is uniquely identifiable according to the Kruskal uniqueness condition (68) for tensor factorization. Specifically, in our data, the number of splicing events is often small and always below the number of subjects (median = 8, as shown in Fig. S7). This yields that the sum of the rank of subject, splicing event, and tissue loading matrices is 2p+t or 3p, whichever is the smaller. This will always be larger than 2p+2, which, according to Kruskal et al.(68), is sufficient to guarantee the uniqueness of the model’s factorization formulation. Second, our approach generates a comprehensive representation of genes’ possible splicing event patterns, ready to be utilized in the downstream sCCA analyses. By aligning r with the number of splicing events, we will be able to represent all possible splicing combinations for each gene (in its splicing event loading matrix). The corresponding subject loading matrix will thus provide a comprehensive profile of each subject’s splicing configurations, for sCCA to effectively extract predictive patterns between SNPs and splicing components. Third, we conducted additional analyses using two alternative rank thresholds: 0.5 and 1.5 times of p, comparing with the results presented here. The results demonstrate that approximately 80% of the genes identified under these alternative thresholds were consistently ranked in the top 3% genes in our primary analysis. These variations in gene identification can be attributed to differences in p-value distributions across the threshold settings. In this way, we ensure that our tensor decomposition is theoretically sound, biologically interpretable, computationally feasible, and optimized for downstream analyses.*”
